# Robust Neutralizing Antibody Responses 6 Months Post Vaccination with BNT162b2: A Prospective Study in 308 Healthy Individuals

**DOI:** 10.3390/life11101077

**Published:** 2021-10-12

**Authors:** Evangelos Terpos, Vangelis Karalis, Ioannis Ntanasis-Stathopoulos, Maria Gavriatopoulou, Sentiljana Gumeni, Panagiotis Malandrakis, Eleni-Dimitra Papanagnou, Efstathios Kastritis, Ioannis P. Trougakos, Meletios A. Dimopoulos

**Affiliations:** 1 Department of Clinical Therapeutics, School of Medicine, National and Kapodistrian University of Athens, 11528 Athens, Greece; johnntanasis@med.uoa.gr (I.N.-S.); mgavria@med.uoa.gr (M.G.); panosmalan@med.uoa.gr (P.M.); ekastritis@med.uoa.gr (E.K.); mdimop@med.uoa.gr (M.A.D.); 2 Section of Pharmaceutical Technology, Department of Pharmacy, School of Health Sciences, National and Kapodistrian University of Athens, 15784 Athens, Greece; vkaralis@pharm.uoa.gr; 3 Department of Cell Biology and Biophysics, Faculty of Biology, National and Kapodistrian University of Athens, 15784 Athens, Greece; sgumeni@biol.uoa.gr (S.G.); epapanagnou@biol.uoa.gr (E.-D.P.); itrougakos@biol.uoa.gr (I.P.T.)

**Keywords:** SARS-CoV-2, COVID-19, neutralizing antibodies, humoral immunity, BNT162b2, vaccine

## Abstract

Elucidating long-term immunity following COVID-19 vaccination is essential for decision-making regarding booster shots. The aim of this study was to investigate the kinetics of neutralizing antibodies (Nabs) against SARS-CoV-2 up to six months after the second vaccination dose with the BNT162b2 mRNA vaccine. Nabs levels were measured on days 1 (before the first vaccine shot), 8, 22 (before the second shot), 36, 50, and 3 and 6 months after the second vaccination (NCT04743388). Three hundred and eight healthy individuals without malignant disease were included in this study. At six months, 2.59% of the participants had a Nabs value less than 30%, while 11.9% had Nabs values of less than 50%. Importantly, 58% of the subjects had Nabs values of more than 75%. Nabs were initially eliminated at a relatively slow rate, but after three months their elimination was 5.7 times higher. Older age was inversely associated with Nabs levels at all examined timepoints. Interestingly, a population modeling analysis estimated that half of the subjects will have Nabs values less than 73.8% and 64.6% at 9 and 12 months, respectively, post vaccination completion. In conclusion, we found a persistent but declining anti-SARS-CoV-2 humoral immunity at six months following full vaccination with BNT162b2 in healthy individuals, which was more pronounced among older persons. These data may inform the public health policies regarding the prioritization of booster vaccine shots.

## 1. Introduction

The coronavirus disease (COVID-19) remains a major public health issue with important implications in all aspects of life. Vaccination campaigns against severe acute respiratory syndrome coronavirus 2 (SARS-CoV-2) are ongoing around the world, with the BNT162b2 mRNA vaccine having a key role in most countries. Although theBNT162b2 is highly efficacious against COVID-19 [1,2,3], a time-dependent decrease in antibody levels against SARS-CoV-2 has been reported among vaccinated individuals [4,5].

The current key question is whether and when a third dose should be administered. The aim of this study was to investigate the kinetics of neutralizing antibodies (Nabs) against SARS-CoV-2 at six months following full vaccination with BNT162b2. BNT162b2 is a mRNA vaccine and induces the production of Nabs in vaccinated individuals. Interestingly, high Nabs levels have been associated with prevention from COVID-19 [6]. The potential impact of subjects’ characteristics including age, gender, co-morbidities, co-medication, and adverse events was also investigated. In addition, a population model was developed to describe the decline of Nabs levels, taking into consideration the possible effect of confounding factors. The model was further used in simulations to predict the Nabs levels at 9 and 12 months after vaccination.

## 2. Materials and Methods

### 2.1. Clinical Procedures

After obtaining approval from the institutional Ethical Committee, the study was conducted at Alexandra General Hospital, Athens, Greece (NCT04743388) [7]. The entire clinical part was in accordance with the Helsinki Declaration and the International Conference for Harmonization for Good Clinical Practice. Before participating in the study, all participants provided informed consent. Vaccination with BNT162b2 mRNA vaccine, age over 18 years, and ability to sign informed consent were among the main inclusion criteria. Potential individuals were excluded if they had active malignant disease or end-stage renal disease, or they were receiving immunosuppressive therapy. Data confidentiality was in accordance with the guidance of General Data Protection Regulation.

### 2.2. Bioanalysis

The blood collection schedules were as follows: day 1 (D1) before the first vaccination, D8, D22 (before the second vaccination), D36, D50, as well as 3 and 6 months after the second vaccination. Within 4 h of blood collection, the serum was separated and stored at −80 °C. SARS-CoV-2 neutralizing antibodies were measured using the FDA-approved cPass^TM^ SARS-CoV2 Nabs Detection Kit (GenScript, Piscataway, NJ, USA).

### 2.3. Statistical Analysis

Statistical analysis was performed on the % inhibition levels (Nabs). To determine the normality of the data distribution, the Kolmogorov–Smirnov and Shapiro–Wilk tests were used. Non-parametric methods were used in the analysis because the Nabs values were found not to follow the normal distribution. The Mann–Whitney U test was used for two independent group comparisons, including the analysis for gender effect. The Wilcoxon test was applied for pairwise group comparisons, including the comparisons of Nabs between two subsequent time points. The Kruskal–Wallis method was used to determine whether there is a difference in Nabs titers among many groups e.g., comparing the three age groups: (20–40), (40–55), ≥55 years. The impact of age, gender, body mass index (BMI), and medical history (i.e., comorbidities) of each subject on the antibody levels at each time point was also examined. The significance level was set at 5%. Python (v.3.9.2) was used for the statistical analysis.

Individual longitudinal % inhibition values were investigated in terms of population kinetic analysis, using the stochastic approximation expectation maximization algorithm for nonlinear mixed effects followed by importance sampling methods [8]. Only the declining part of the Nabs levels was modeled, since the focus of this study was on assessing the rate of Nabs elimination from the body. Thus, data from two weeks after the second vaccination up to six months were utilized.

Several structural models like one- and two-compartment were assessed. Nabs elimination kinetics were modelled by single exponential or linear functions as well as piecewise (exponential or linear) functions. Nabs levels were considered either normal or log-normal, while several residual error models were tested (e.g., constant, proportional, and combined). After the development of the final best structural model, the individuals’ characteristics (e.g., age, gender) were tested for the impact on the model parameters. The Wald test was used to determine whether covariates could explain parameter variation. The entire computational work was carried out by writing the necessary code in Monolix^TM^2020R1 Mlxtran language (Lixoft, Orsay, France, Simulation Plus). After the development of the kinetic population model, simulations were performed to predict Nabs concentration levels 9 and 12 months after vaccination, assuming that the kinetic parameters remain unchanged after the third month. Simulx^®^ (Lixoft, Orsay, France, Simulation Plus) was used for the simulations.

## 3. Results

This study involved 308 individuals who received two doses of BNT162b2 mRNA vaccine. Nabs levels were measured at day 1, 8, 22, and 2 weeks, 4 weeks, 3 months, and 6 months after the second vaccination in all. Table 1 shows the demographic data of the subjects who participated in this study. The mean age was 48.1 years, and nearly two-thirds of the participants were women.

**Figure 1:**
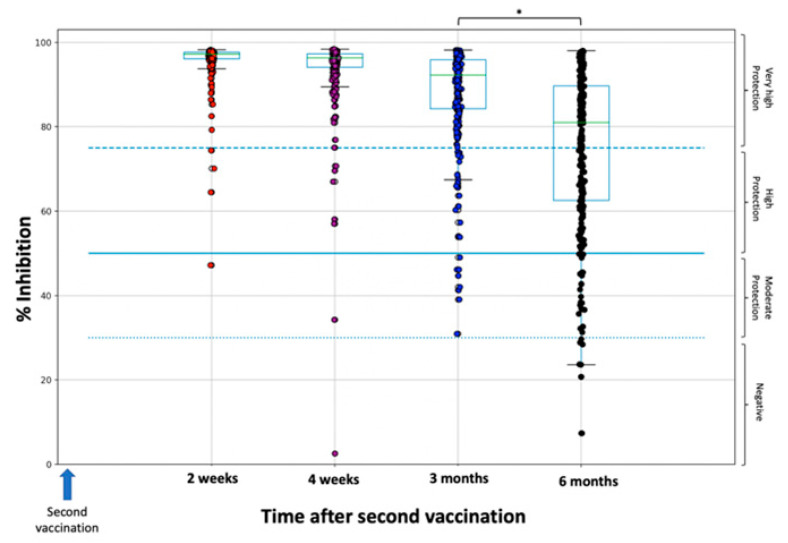
Inhibition (%) of SARS-CoV-2 binding to the human host receptor angiotensin converting enzyme-2 for the whole study population. The asterisk (*) indicates statistically significant differences (*p*-value < 0.05) between the groups. The boxplot borders correspond to the quartiles of the distribution. Each dot represents each individual value of Nabs inhibition. Dashed lines show the borderline levels of inhibition (30%, 50%, and 75%).

**Figure 2:**
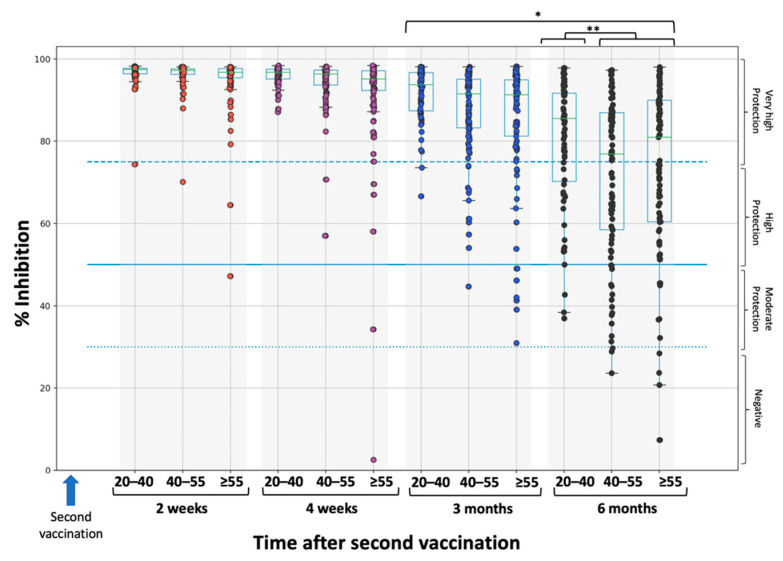
Inhibition (%) of SARS-CoV-2 binding to the human host receptor angiotensin converting enzyme-2 for the three age groups: 20–40, 40–55, and ≥55 years. Asterisks (* or **) indicate statistically significant differences (*p*-value < 0.05) between the compared groups. The boxplot borders correspond to the quartiles of the distribution. Each dot represents each individual value of Nabs inhibition. Dashed lines show the borderline levels of inhibition (30%, 50%, and 75%).

Figure 1 and Figure 2 show the percentage inhibition of Nabs on each day of measurement for the whole study population (Figure 1) and divided by age groups: 20–40, 40–55, and ≥55 years (Figure 2). Since the focus of this study was to describe the elimination kinetics of Nabs, only the results from two weeks after the second vaccination, when maximum inhibition is achieved, are shown. At that time point, the median overall inhibition is 97.2% (specifically 97.5% for the 20–40-year-old group, 97.2% for the 40–50-year-old group, and 96.8% for the ≥55-year-old group). It is also clear that as age increases, the dispersion towards lower inhibition values becomes larger. After this time point, Nabs values decrease steadily for up to six months. During this decline period, it is evident that the greater Nabs dispersion to lower values increases with age.

At three months and six months, the median inhibition values were 92.3% and 81.0%, respectively. At three months, there were no subjects with Nabs below the 30% threshold. At six months, the proportion of subjects with a Nabs value of 30% was 2.59% (8 subjects), while 11.9% of participants had Nabs values of less than 50%. It is noteworthy that a proportion of 58% of the subjects had Nabs values of more than 75%. A significant difference (*p* < 0.001) was found between three and six months (Figure 2). Regarding the effect of age with Nabs less than 30%, there was no subject in the 20–40 age category, while the proportions in the 40–55 and ≥55 groups were 3.16% and 4.49%, respectively. Notably, for subjects above 60 years of age (data not shown), the proportion further increases to 5.66%. A statistically significant difference (*p* = 0.018) was found between the 20–40 group and the other two groups.

**Figure 3:**
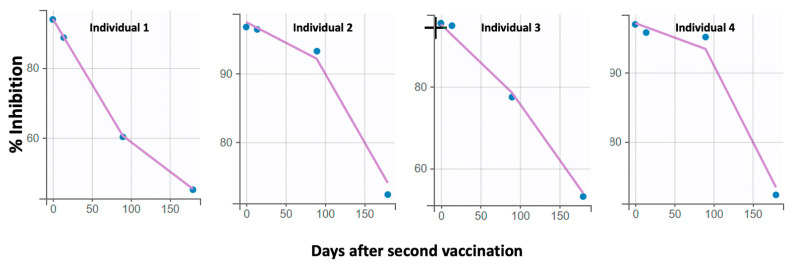
Representative individual profiles of Nabs values (% inhibition) versus time. The solid lines refer to the values predicted by the model, while the circles represent the observed Nabs levels. The good descriptiveness of the model is expressed by the close passage of the line predicted by the model to the actual points.

**Figure 4:**
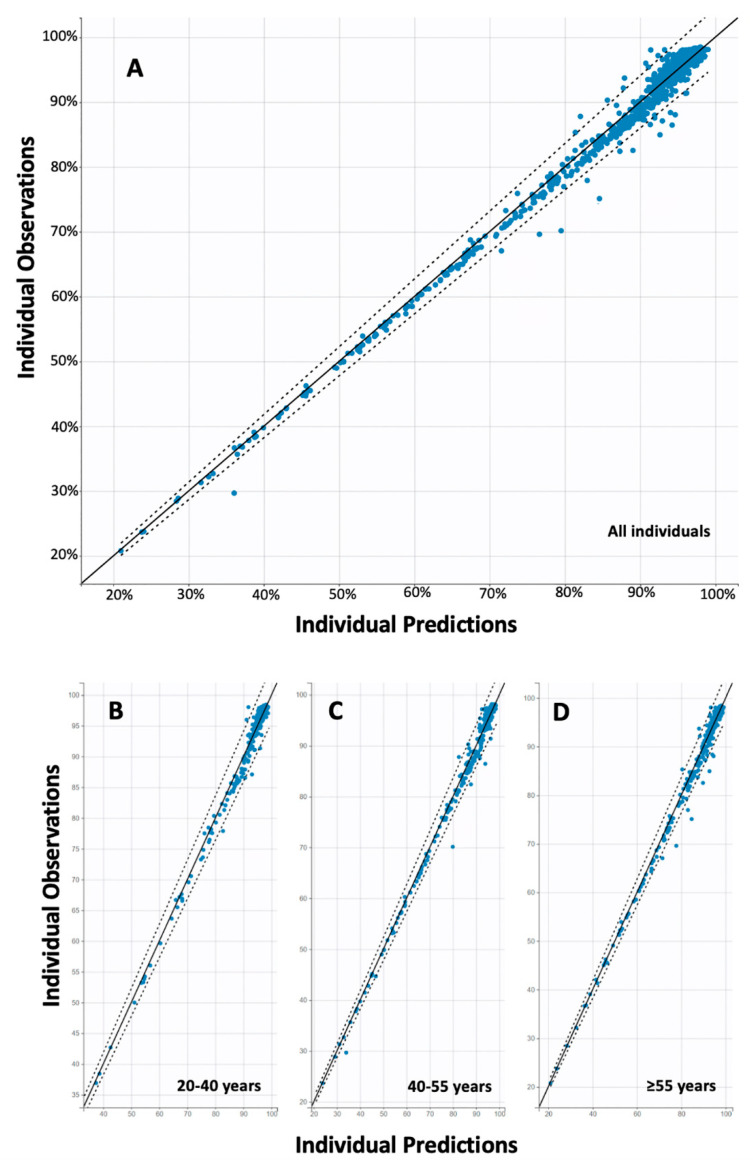
Individual observed versus predicted nabs values. Total goodness-of-fit results are shown for: (**A**) the whole data, (**B**) the age group of 20–40 years, (**C**) of 40–55 years, and (**D**) the individuals of ≥55 years. The solid line is the dichotomous line referring to the ideal prediction performance, while the dashed line indicates the 90% prediction interval.

Furthermore, the final best model derived from the population kinetic analysis included a piecewise function for the elimination constant and a proportional error model. The first elimination constant (*kel1*) was determined to be 0.017 Nabs/day (standard error = 0.002) and referred to the initial decay phase up to three months. Thereafter, the elimination constant was *kel2* = 0.097 Nabs/day (standard error = 0.007). This result means that Nabs are initially eliminated at a relatively slow rate, but after three months their elimination is 5.7 times higher, which means that they are eliminated faster. Age was found to affect both phases of elimination, with older age leading to faster elimination of Nabs. However, this relationship was statistically significant (*p* < 0.001) only in the initial phase (up to three months). The mathematical model describing the effect of age on the elimination constant is expressed as follows: *log(kel1)* = *log(kel1_pop)* + *eta_kel1*, where *kel1_pop* and *eta_kel1* refer to the average and random effect for inter-subject variability. No other factors such as gender, BMI, etc. were found to have a statistically significant effect on antibody kinetics. A positive correlation (coefficient = 0.84) was found between *kel1* and *kel2*. The goodness of fit and validation plots of the final model are shown in Figure 3 and Figure 4.

**Figure 5:**
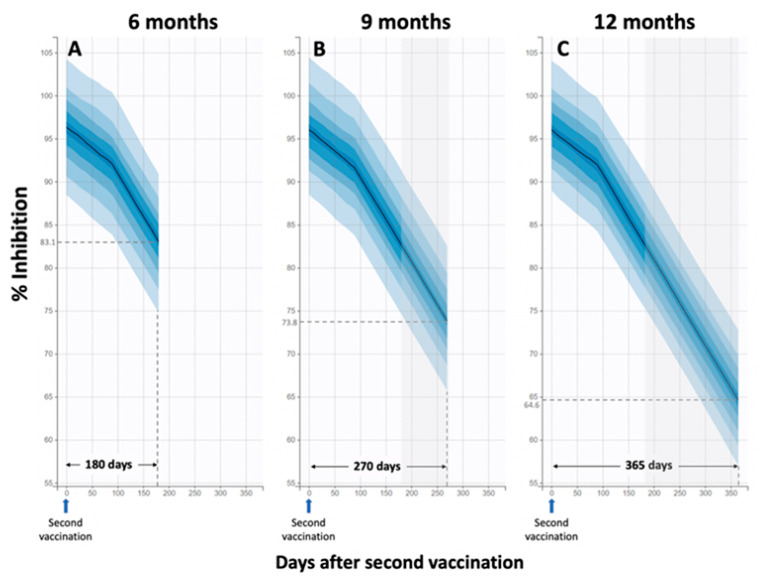
Observed and simulated Nabs levels over a period of several months. Simulations were performed for 6 months (**A**), 9 months (**B**), and 12 months (**C**) after the second vaccination. The first elimination constant (kel1) was determined to be 0.017 Nabs/day (standard error = 0.002) and referred to the initial decay phase up to 3 months. Thereafter, the elimination constant was kel2 = 0.097 Nabs/day (standard error = 0.007). Simulated inhibition (%) levels versus time for three different time frames: six, nine and twelve months. At 6 months, the predicted median of 83.1% is quite close to the observed median of 81.0%. For the predictions 9 and 12 months after injection, it is assumed that the kinetic parameters remain the same as observed in the interval 3–6 months.

Interestingly, simulations were performed using the final population model to predict Nabs levels at 9 and 12 months after the second vaccination, assuming that the elimination of Nabs would be the same as that observed at 3–6 months. These simulations show that the median percent inhibition at 9 and 12 months will be 73.8 and 64.6, respectively. The more rapid decline after three months is also evident (Figure 5).

## 4. Discussion

Our results are consistent with another prospective study including 122 individuals vaccinated with BNT162b2 [9]. A waning humoral response against SARS-CoV-2 was evident at three and six months post vaccination, especially among older individuals [9]. A persistent but declining antibody activity at six months post vaccination has been also reported for the mRNA-1273 [10,11]. Importantly, results from clinical trials have shown that BNT162b2 offers a robust protection from COVID-19 (91.3%, 95%CI: 89.0–93.2) at 6 months following vaccination [12].

Age has emerged as one of the most important predictive factors for the quality of antibody response following both vaccination and recovery from COVID-19 [7,13,14,15]. This may be associated with the ability of inducing robust immune responses after exposure to SARS-CoV-2 antigens [14].

It has to be mentioned that several other factors beyond age may be responsible for the variability in the sustainability of humoral responses against SARS-CoV-2 post vaccination, similar to the variable interindividual patterns of antibody response following recovery from COVID-19 [16]. The sustainability of T-cell responses following vaccination is another factor to consider. BNT162b2 induces robust T-cell responses against both wild-type SARS-CoV-2 and variants of concern for at least three months following vaccination [9,17,18].

The importance of our findings lies in the predictive value of Nabs levels regarding the immune protection from symptomatic COVID-19 [6]. Therefore, the described Nabs reduction in time may inform public health policies in order to implement booster doses. Although emerging SARS-CoV-2 variants of concern challenge the high rate of immune protection following vaccination [19], a booster vaccine shot along with transmission-reducing behaviors remain effective in preventing COVID-19 [20,21]. It should be emphasized that in our simulations we used the same kinetic parameter values for the extrapolation period after six months as were found in the interval of three to six months. This choice was made since it is considered the safest for predictions, as there are no findings on kinetics after six months from either the data or the literature. Thus, this choice would avoid large over- or underestimates of Nabs values.

A limitation of the study was the relatively small sample size, which hindered the investigation of specific pathophysiological conditions such as autoimmune diseases. Further studies could also evaluate the neutralization efficacy of anti-SARS-CoV-2 antibodies against variants of concern.

## 5. Conclusions

This study examined the kinetics of Nabs against SARS-CoV-2 in 308 individuals after vaccination with BNT162b2 mRNA vaccine. Six months after the second vaccination, 2.8% of individuals have a Nabs value of less than 30%, while this proportion increases to approximately 6% in older individuals (60 years). Only age was identified as a significant factor affecting elimination of Nabs. The population kinetic model showed that elimination of Nabs is biphasic, with a relatively slow phase up to three months and a much faster phase (more than five-fold) after month three. Simulations showed that after nine months, half of the subjects are expected to have Nabs titers of less than 73.8%, while one year after the second vaccination, half of the subjects will have Nabs titers of less than 64.6%. These results provide important insights into the timing of potential booster vaccinations, which may be age-dependent.

## Figures and Tables

**Table 1:** Baseline characteristics of study participants.

Characteristics	Value
Number of participants	308
Women (n, %)	202 (65.6%)
Men (n, %)	106 (34.4%)
Age (median, range) (years)	48.1 (49)
20–30 (n, %)	34 (11.0%)
30–40 (n, %)	58 (18.8%)
40–50 (n, %)	75 (24.4%)
50–60 (n, %)	77 (25.0%)
≥60 (n, %)	64 (20.8%)
BMI (median)	25.1
Underweight (n, %)	14 (4.5%)
Normal weight (n, %)	148 (48.1%)
Overweight (n,%)	102 (30.8%)
Obese (n, %)	44 (13.6%)
PCR+ (n, %)	22 (7.14%)

n, number of subjects; BMI, body mass index; PCR+, prior history of positive PCR test for SARS-CoV-2.

## Data Availability

The data presented in this study are available on request from the corresponding author.
